# Research Trends and Hotspots of Q Fever Research: A Bibliometric Analysis 1990-2019

**DOI:** 10.1155/2022/9324471

**Published:** 2022-01-15

**Authors:** Muhammad Farooq, Aman Ullah Khan, Hosny El-Adawy, Katja Mertens-Scholz, Iahtasham Khan, Heinrich Neubauer, Yuh-Shan Ho

**Affiliations:** ^1^Department of Clinical Sciences, College of Veterinary and Animal Sciences, Jhang, University of Veterinary and Animal Sciences, Lahore, 35200 Jhang, Pakistan; ^2^Department of Pathobiology, College of Veterinary and Animal Sciences, Jhang, University of Veterinary and Animal Sciences, Lahore, 35200 Jhang, Pakistan; ^3^Institute of Bacterial Infections and Zoonoses, Friedrich-Loeffler-Institut, 07743 Jena, Germany; ^4^Faculty of Veterinary Medicine, Kafrelsheikh University, Kafr El-Sheikh 35516, Egypt; ^5^Trend Research Centre, Asia University, No. 500, Lioufeng Road, Wufeng, Taichung 41354, Taiwan

## Abstract

Q fever is a worldwide distributed zoonosis caused by *Coxiella burnetii*, a Gram-negative bacterium. Despite existence of large amount of research data on the developments related to Q fever, no bibliometric analysis of this subject is available to our knowledge. Bibliometric studies are an essential resource to track scholarly trends and research output in a subject. This study is aimed at reporting a bibliometric analysis of publications related to Q fever (2,840 articles published in the period 1990-2019) retrieved from Science Citation Index Expanded, an online database of Clarivate Analytics Web of Science Core Collection. Data was retrieved using keywords “Q fever” or “*Coxiella burnetii*” in title, abstract, and author keywords to describe important research indicators such as the kind and language of articles, the most important publications, research journals and categories, authors, institutions, and the countries having the most significant contribution to this subject. Finally, the emerging areas in field of diagnosis, host range, and clinical presentation were identified. Word cluster analysis of research related to Q fever revealed that major focus of research has been on zoonosis, seroprevalence, laboratory diagnosis (mainly using ELISA and PCR), clinical manifestations (abortion and endocarditis), vectors (ticks), and hosts (sheep, goat, and cattle). This bibliometric study is intended to visualize the existing research landscape and future trends in Q fever to assist in future knowledge exchange and research collaborations.

## 1. Introduction

Q fever is recognized as a global zoonotic disease that has been declared as potential bioterrorism category B select agent by the Centre for Disease Control and Prevention (CDC) [1]. It is regarded as a reportable disease in some countries [2]. This disease is caused by *Coxiella burnetii*, an obligate Gram-negative bacterium, which can infect human, various animals such as ruminants (cattle, goat, and sheep), pets, birds, ticks, and rarely reptiles and marine mammals [3]. This bacterium is secreted in birth products (such as placenta), urine, milk, and faeces [3, 4]. Main route of transmission is inhalation of contaminated aerosols. However, ingestion of contaminated raw milk can at least cause seroconversion. Human-to-human transmission was described and might happen through contaminated blood transfusion, sexual contact, and exposure to contaminated birth products of women. Mainly, this disease is reported in humans having close contact with infected animals and their products [3].

Q fever can manifest as an acute or chronic disease. Acute infections are mostly asymptomatic (60%) or manifests as a flu-like and often self-limiting disease. Symptoms include but are not limited to flu-like symptoms, endocarditis, hepatitis, pneumonia, abortion, and premature fetal death in pregnant women and neuropathies [5]. Differentiation of acute from chronic Q fever solely on clinical manifestation may be misleading. Currently, acute and chronic forms are differentiated on the basis of different antibodies present in the sera of the patient. This demonstrates that presence of IgG to phase I indicates the chronic form while detection of IgG to phase II antigen demonstrates acute form [6]. In most of the cases, it is asymptomatic and therefore remains underreported. Different techniques are used for its diagnosis such as IgG-based serological assays and DNA-based molecular assays [5]. In symptomatic patients seeking medical advice, this disease can be treated through administration of antibiotics such as doxycycline [3].

Despite existence of large amount of research data on Q fever, to best of our knowledge, no bibliometric analysis of this topic is available. Bibliometrics makes it easy to investigate and decipher different developments on a subject to pursue the dynamics and evolution of scientific knowledge. Identifying future research directions based on a bibliometric analysis of the characteristics of available literature in a field reduces the error margin and thus improves the decision-making. These indicators may further be helpful for early career researcher to identify the latest developments of the topic.

The present bibliometric study is intended to analyze 3,673 Q fever-associated publications retrieved from the Science Citation Index Expanded (SCI-EXPANDED) database of Web of Science, the most-acknowledged database in bibliometric studies [7]. The retrieved data were analyzed according to language and type of publication, most productive authors, laboratories, countries, and scientific journals as well as the most cited articles. Based on this analysis, hotspots and recent trends in scientific developments pertinent to Q fever were identified and discussed in this study.

## 2. Methodology

This study relied on the data obtained from Clarivate Analytics Web of Science Core Collection (WoSCC), a platform of the SCI-EXPANDED. All data were obtained on February 2021 by searching the database for articles related to Q fever with the following parameters: WoS TOPIC (title, abstract, author keywords, and *KeyWords Plus*): (“Q fever” or “*Coxiella burnetii*”) and year (1990-2019). Use of quotation marks (“ ”) is essential to identify the exact searched terminology by avoiding the lemmatization and synonym features of WoSCC (by default, in search setting, these features are ON) [7]. Owing to this search feature, it was necessary to find different expressions, and therefore, Boolean operator “or” was used which ensured the appearance of at least one term (“Q fever” or “*Coxiella burnetii*”) in the topic.

Database search by using this strategy yielded 4,270 documents including 3,242 articles. It is important to note that additional search terms can be obtained by *KeyWords Plus* which are extracted from article titles enlisted as references (in reference list or footnotes) in the Clarivate Analytics database which causes a substantial increase in title-word and author-keyword indexing [8]. SCI-EXPANDED has been widely used for bibliometric studies, but it is mainly designed to facilitate authors to search suitable literature but not meant for direct bibliometric analyses [9]. Therefore, to avoid inclusion of irrelevant documents to the desired ones, use of SCI-EXPANDED necessitate the accurate bibliometric analysis instead of relying only on *KeyWord Plus* [10]. Therefore, use of “front page” (that considers the abstract, manuscript title, and author keywords) has been suggested as used as a filter [11]. This filter has been employed in present study by considering only the search keywords which were found in their “front page.” This modified method by considering “front page” as a filter resulted in 3,673 documents (86% of 4,270). These documents were used for further analysis.

Impact factor of journals (mentioned as IF_2019_) was obtained from the Journal Citation Reports in 2019. After downloading, the data were arranged using Microsoft Excel 2016 as described elsewhere [12]. The term of “corresponding author” (*RP*) was used though it is designated as “reprint author” in SCI-EXPANDED database [13]. Single author in articles with unspecified authorship was both the first as well as corresponding author. Similarly, articles published by single institution were classified as the institution of the first author and corresponding author [13]. Moreover, in articles having multiple corresponding authors, we only considered the last corresponding author. Type of collaboration was assessed by using addresses of the authors. Geographic location of the countries was determined as described elsewhere [7].

Publication citations were assessed using following indicators:

- C_year_. The number of citations from WoSCC in a particular year (e.g., C_2019_ describes citation count in 2019) [13]
- TC_year_. The total citations from WoSCC received since publication year till the end of the most recent year (2019 in this study, TC_2019_) [14]
- CPP_year_. Citations per publication (CPP_2019_ = TC_2019_/TP), TP: total number of publications [13]

## 3. Results and Discussion

### 3.1. Characteristics of Document Types

It has been recently suggested to identify the characteristics of a document type on the basis of their citations per publication (CPP_year_ = TC_year_/TP) and number of authors per publication (APP = AU/TP) [15]. Use of TC_2019_ for CPP_2019_ is advantageous owing to their invariability and ensured repeatability as compared to the citation index from WoSCC [16]. A total of 3,673 Q fever related documents published in SCI-EXPANDED were found among 12 document types which are listed in Table 1. This publication count includes 2,840 (77%) articles having 6.0 as APP (number of authors per publication) which was higher than most other document types except reprint with an APP value of 16. The percentage of articles focusing on Q fever (77%) was higher than other medical-related topics, for example, 70% in Ebola [17], cisplatin-based chemotherapy for small cell lung cancer (68%) [18], and acupuncture (66%) [19] but similar to 75% human papillomavirus [20] and 79% in asthma in children [21]. The document type “review” with 217 documents had the greatest CPP_2019_ value of 53 followed by articles with CPP_2019_ of 20. The CPP_2019_ of the document type “review” was found to be 2.6 times of articles. Six of the top ten documents in *TC*_2019_ were reviews in Q fever research including “Q fever” with TC_2019_ of 1,220 [22], “Ticks and tickborne bacterial diseases in humans: An emerging infectious threat” with TC_2019_ of 585 [23], “Antimicrobial mechanisms of phagocytes and bacterial evasion strategies” with TC_2019_ of 485 [24], “Natural history and pathophysiology of Q fever” with TC_2019_ of 434 [25], “Endocarditis due to rare and fastidious bacteria” with TC_2019_ of 398 [26], and “Diagnosis of Q fever” with TC_2019_ of 372 [27].

It is important to point out that documents could be categorized in two document types in WoS. For example, the highly cited document entitled “Comparative study of the clinical presentation of *Legionella pneumonia* and other community-acquired pneumonias” [28] was classified as “article” and “proceedings paper.” Therefore, cumulative percentages exceed 100%.

Among publication types, only “articles” (2,840) among different document types were chosen for further analyses due to the presence of original research ideas and results therein [29]. These articles were presented in 14 different languages. English, with 92% of the 2,840 Q fever articles, was the most used language followed by German (83 articles), French (67 articles), Spanish [29], Russian [14], Dutch [10], Czech [7], Italian [6], Hungarian [5], Turkish [5], Portuguese [4], Polish [3], Japanese [2], and Ukrainian [1]. Non-English articles had less citations, with CPP_2019_ of 5.0, while English articles had CPP_2019_ of 22.

### 3.2. Characteristics of Publication Outputs

**Figure 1:**
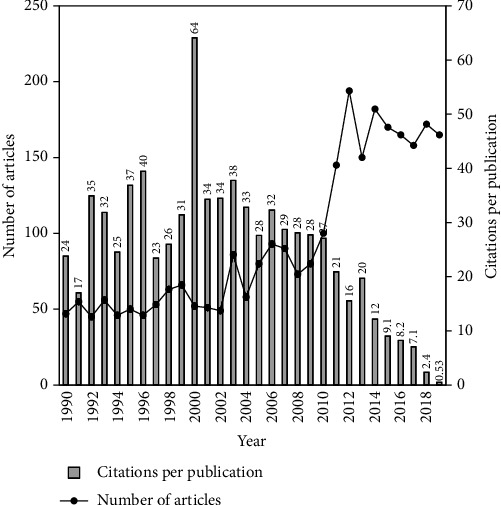
Number of highly cited articles and citations per publication by year.

To determine CPP_2019_, use of TC_2019_ was found better than citation index from WoSCC directly because they are consistent and ensure repeatability [30]. To better understand the trends and impacts of publications in a particular research theme, Ho established a correlation between total articles (TP) in a year and their citations (CPP_year_ = TC_year_/TP) by the decades [13] and years [18]. Recently, it has been applied in medical-related topics, for example, pain [31], Ebola [17], and dengue [32]. Between 1990 and 2019, 2,840 articles associated to Q fever were published. The mean value of TC_2019_ was 20 with 1,903 as the maximal value for an article.  Figure 1 demonstrates the distribution of the annual number of articles and their citations per publication (CPP_2019_) by year, which was expressed as TC_2019_/*TP* [33], where TP is number of articles published in that particular year. The number of articles sharply increased from two in 2008 to reach a peak of 194 in 2012. Fifty-two articles published in 2000 had the highest CPP_2019_ of 64 which can be attributed to two of the top five most frequently cited articles by Li et al. (2000) with TC_2019_ of 1,903 (ranked 1st) and Raoult et al. (2000) with TC_2019_ of 342 (ranked 5th). Based on Figure 1, citations per publication related to Q fever articles attained a plateau in 10 years of publication. Similarly, one decade was taken to attain a plateau by articles related to dengue [32] and Ebola [17].

### 3.3. Web of Science Category and Journal

Journal Citation Reports (JCR) indexed 9,381 journals with citation references across 178 Web of Science categories in SCI-EXPANDED in 2019. In order to know development among research fields and their interactions, Ho et al. proposed a relationship between the number of articles in categories and publication years [29]. Total 615 journals published articles related to Q fever in 87 WoS categories in SCI-EXPANDED. Following five categories were highly productive with total number of 2,018 articles (71% of 2,836 articles) including (i) infectious diseases with 902 articles (32% of 2,836 articles), (ii) microbiology with 739 (26%) articles, (iii) veterinary sciences with 533 (19%), (iv) immunology with 499 (18%) articles, and (v) public, environmental, and occupational health with 350 (12%) articles. Similar to articles, journals can also be listed in more than one category in WoSCC like Clinical Infectious Diseases belongs to multiple categories (immunology and infectious diseases and microbiology). Therefore, cumulative percentage of categories exceeds 100% [34]. In total of 144 articles (rank 9th) were published in category of parasitology including 16 articles (rank 6th) in 2019.

In total, 2,840 Q fever-related articles were published in 615 journals including 539 listed in SCI-EXPANDED in 2019.  Table 2 provides the list of the top 10 most productive journals. PLoS One (IF_2019_ = 2.74) published the most articles (104) which represent 3.7% of 2,840 articles. Q fever articles published in Clinical Infectious Diseases (IF_2019_ = 8.313) had the highest CPP_2019_ of 76 while articles in Acta Virologica (IF_2019_ = 0.793) had CPP_2019_ of 8.6 (Table 2). It showed a positive relationship between IF_2019_ and CPP_2019_. Five of the top ten productive journals belong to the category of infectious diseases. The number of authors ranged from 4.6 in Acta Virologica and European Journal of Epidemiology, respectively, to 7.9 in the American Journal of Tropical Medicine and Hygiene indicating most papers required multiple authors. The journal with the highest IF_2019_ of 74.699 was New England Journal of Medicine followed by Lancet (IF_2019_ = 60.390) and *Science* (IF_2019_ = 41.846) with one article, respectively.

### 3.4. Publication Performances: Countries and Institutions

In order to evaluate publication output of institutions and countries, Ho's group proposed six publication indicators including the total number of publications (TP), first-author publications (FP), single-author publications (SP), corresponding-author publications (RP), independent publications (IP), and collaborative publications (CP) [29]. First and the corresponding authors are widely recognized for having most of the contribution in an article [35]. At the institutional level, the determined institution of the corresponding author might be the hosting institution or university of the study or origin of the paper [13]. There were 16 articles (0.56% of 2,840 articles) without affiliations in SCI-EXPANDED. Q fever articles (2,824) were published by authors affiliated from 121 countries. However, 2,216 articles (78% of 2,824 articles) were published from 65 countries by authors affiliated from single country, while remaining 608 articles (22% of 2824) were published by authors from 117 countries as internationally collaborative articles. This situation demonstrates that collaborative research is very limited and should be focused on future studies.

**Figure 2:**
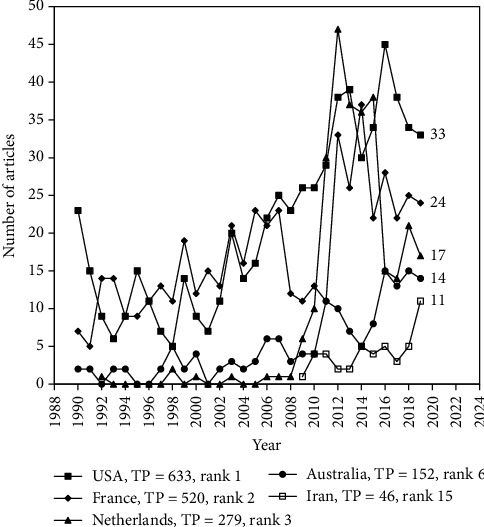
Developments of the top five productive countries in 2019.

Table 3 enlists the top 10 most productive countries with six publication indicators [36] and a citation indicator (CPP_2019_). Use of CPP_2019_ is advantageous due to its invariant nature as compared to the citation index from the WoSCC which is regularly updated [11]. The USA was the top-ranking country in the six publication indicators with TP of 633 articles (22% of 2,824 articles), IP of 434 articles (20% of 2,216 independent articles), CP of 199 articles (33% of 608 internationally collaborative articles), FP of 517 articles (18% of 2,824 first-author articles), RP of 506 articles (19% of 2,733 corresponding-author articles), and SP of 29 articles (32% of 92 single-author articles). France had the highest CPP_2019_ of 30 while Germany, Spain, and Netherland had only 16. Among the 121 countries published Q fever articles, 56 countries (42% of 121 countries) had no single country articles while four countries (3.3%) had no internationally collaborative articles. Similarly, 32 (26%), 37 (31%), and 95 (79%) countries have no first-author, corresponding-author, and single-author articles, respectively. Trends in publication of the top five productive countries in 2019 are presented in Figure 2. Iran had TP of 46 (ranked 15th) and was a new member of productive countries with 11 articles in 2019.

With regard to institutions, 919 Q fever-related articles (33% of 2,824 articles) originated from single institutions while 1,905 articles (67%) were institutional collaborations. List of top 10 productive institutions and their characteristics are presented in Table 4. Four of them were located in France, three in the Netherlands, two in the USA, and one in Slovakia.

The Aix Marseille University in France took the leading position for total articles with TP of 104 articles (3.7% of 2,824 articles). The University of Mediterranee in France ranked top in three of the six publication indicators with IP of 39 articles (4.2% of 919 in single institution articles), FP of 75 articles (2.7% of 2,824 first-author articles), and RP of 69 articles (2.5% of 2,733 corresponding-author articles). Jeroen Bosch Hospital in Netherlands ranked top in interinstitutionally collaborative articles with CP of 95 articles (5.0% of 1,905 interinstitutionally collaborative articles). In addition, Dalhousie University in Canada published 30 Q fever articles (ranked 19th) including the most single-author articles with SP of five articles (5.4% of 92 single-author articles). The Faculte de Medecine Marseille in France had the highest CPP_2019_ of 57 followed by University of Mediterranee in France with CPP_2019_ of 51. Aix Marseille University in France and Radboud University of Nijmegen in Netherlands had lower CPP_2019_ of 12, respectively.

### 3.5. Publication Performances: Authors

**Figure 3:**
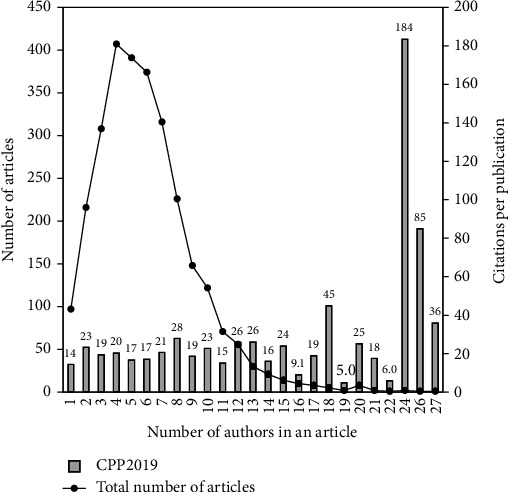
Number of articles and citations per publication by number of authors in an article.

For articles related to Q fever, average number of authors per Q fever article was 6.0 whereas maximum number of authors was 27 in one article. Of the 2,837 articles with author information, 407 (14%), 391 (14%), and 374 (13%) were written by groups of 4, 5, and 6 authors, respectively.  Figure 3 shows the relationship among number of articles (TP) and citations per publication (CPP_2019_) by number of authors in an article. There were two articles which were published by 24 authors [37, 38] including the one with the highest CPP_2019_ of 184 entitled “Complete genome sequence of the Q-fever pathogen *Coxiella burnetii*” having the highest TP_2019_ of 364 [38].

Table 5 lists the top 10 productive authors, among whom, D. Raoult was the only one published single-author articles. In addition, T.J. Marrie published the most of single-author article with seven articles (7.2% of 97 single-author articles). Publication performance of authors was further analyzed. In recent years, the *Y*-index was suggested [13, 33, 34] to evaluate potential of publications and to characterize the scientific publications by authors, institutes and respective countries to the number of articles as first author (FP) and as corresponding author (RP). Recently, the *Y*-index has been demonstrated in research publications in medical research such as highly cited articles in health sciences and dentistry [39]. With two parameters (*j*, *h*), the *Y*-index can be helpful in visualizing and comparing among different author's publications and is described as:

(1) $$\begin{array}{c} j=\text{FP}+\text{RP}, \end{array}$$

(2) $$\begin{array}{c} h=\tan^{-1}\left(\frac{\text{RP}}{\text{FP}}\right). \end{array}$$

**Figure 4:**
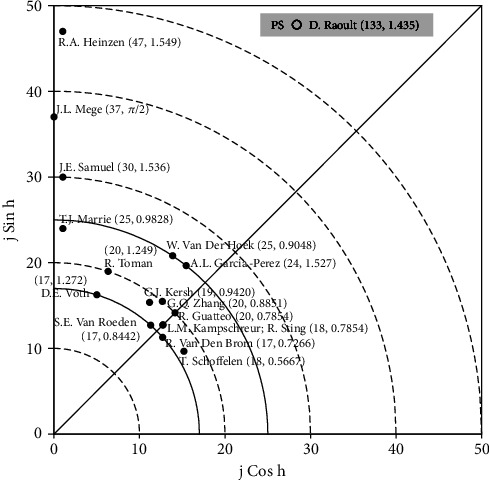
Top 17 authors with *Y*-index (*j* ≥ 17).

In *Y*-index diagram (Figure 4), the authors with higher *j* value are situated further away from origin of the polar coordinates (0, 0). Authors having similar number of articles as first and corresponding author would have *h* = 0.7854 (radian) and located in diagonal line. Furthermore, with more publications as corresponding author as compared to that as first author demonstrating *h* > 0.7854, the author would be represented in the upper left half quadrant of the *Y*-index diagram. However, an author having higher number of publications as first author than as corresponding author, with *h* < 0.7854, would be represented positioned in lower right half of the quadrant. The authors with *h* = 0 and *j* = number of first-author articles then would be positioned along *x*-axis of the diagram. However, when *h* = *π*/2 then *j* = number of corresponding-author articles, the author would be demonstrated along the *y*-axis of the diagram.

In total of 2,683 Q fever articles (95% of 2,840 publications), both as first and corresponding-authors in SCI-EXPANDED were extensively investigated based on *Y*-index. The 2,683 Q fever-related articles were contributed by 9,168 authors in which 6,934 authors (76% of 9,168 authors) had no first- or corresponding-author articles with *Y* − index = (0, 0); 439 (4.8%) authors published only corresponding-author articles with *h* = *π*/2; 99 (1.1%) authors published more corresponding-author articles with *π*/2 > *h* > 0.7854; 944 (10%) authors published the same number of first- and corresponding-author articles with *h* = 0.7854; 80 (0.87%) authors published more first-author articles with 0.7854 > *h* > 0; and 672 (7.3%) authors published only first-author articles with *h* = 0. In Figure 4, distribution of the *Y*-index (*j*, *h*) of the leading 17 potential authors with *j* ≥ 17 was demonstrated. Every point has a coordinate (*j*, *h*) that could symbolize a single-author or multiple authors, for example, L.M. Kampschreur and R. Sting with the same *Y*-index (18, 0.7854). D. Raoult (133, 1.435) published 284 Q fever-related articles which includes 16 articles as first-author and 117 articles as corresponding-author with *j* value of 133 which is far away from the original (not in Figure 4). D. Raoult had the highest publication potential in Q fever research. It is worth mentioning that D. Raoult is a renowned infectious disease expert. He cultured 16% of the newly isolated bacteria from human [3]. Moreover, he has published (Figure 4) the most corresponding-author articles as revealed by high *h* value (1.460).

R.A. Heinzen (42, 1.546) from National Institute of Allergy and Infectious Diseases (NIAID), USA, ranked second in publication potential with *j* value of 47 followed by J.L. Mege [37] from the APHM (Assistance Publique-Hopitaux de Marseille) in France who published corresponding-author articles only followed by J.E. Samuel from Texas A&M University in the USA with a *j* of 30. Only five of the top ten productive authors ranked top ten in *j* including D. Raoult, J.L. Mege, R.A. Heinzen, W. van der Hoek, and J.E. Samuel who are not only productive authors but also in important role in Q fever research (Figure 4). D.E. Voth (17, 1.272), S.E. Van Roeden (17, 0.8442), and R. Van Den Brom (17, 0.7266) all had the same *j* of 17. All these authors are located on the same curve (*j* = 17) in Figure 4, indicating that they had the same publication potential with a *j* of 17 but different publication characteristics [30]. Both published more corresponding-author articles with an *h* of 1.272 then Van Roeden with an *h* of 0.8442. However, Van Den Brom published more first-author articles with an *h* of 0.7266. Similarly, T.J. Marrie (25, 0.9828) and W. Van Der Hoek (25, 0.9048); R. Toman (20, 1.249), G.Q. Zhang (20, 0.8851), and R. Guatteo (20, 0.7854); and L.M. Kampschreur (18, 0.7854), R. Sting (18, 0.7854), and T. Schoffelen (18, 0.5667) are also located on the same curve with *j* of 25, 20, and 18, respectively. R. Guatteo (20, 0.7854), L.M. Kampschreur (18, 0.7854), and R. Sting (18, 0.7854) are located on the diagonal line (*h* = 0.7854). Guatteo had the greatest publication potential with a *j* of 20 followed by Kampschreur and Sting with a *j* of 18. Thus, the location on the graph along one of the curves or along a line from the origin represents different families of author publication potential or publication characteristics, respectively. It has been pointed out that with these data have a potential for bias in the analysis of authorship; it might attribute to different authors having the same name or the same author using different names over time [30].

### 3.6. Citation Histories of the Ten Most Frequently Cited Articles

Total citations are updated weekly on the WoSCC. To improve bibliometric study, the total number of citations from the WoSCC since publication to the end of the most recent year of 2019 (TC_2019_) was applied to improve the bias using data from WoS directly. Use of TC_2019_ is advantageous because of their invariability and ensured repeatability than the citation index from WoSCC [11]. The 2,840 Q fever articles were selected with search keywords within title, abstract, and author keywords from SCI-EXPANDED in the last three decades. A total of 2,104 articles (74% of 2,840 articles); 2,601 articles (92% of 2,686 articles with abstract); and 1,310 articles (46% of 1,720 articles with author keywords) contained the search keywords in their title, abstract, and author keywords, respectively. The title of an article states the article subjects [40].

**Figure 5:**
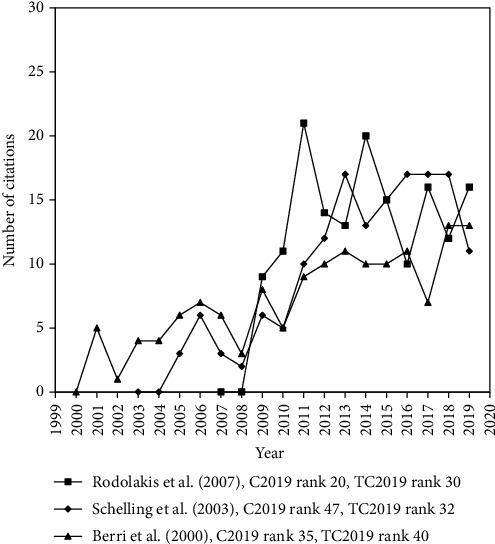
The citation histories of the three highly cited articles.

Author keywords were given by authors to offer more information about the main research focus of their article. Articles that contain search keywords in their abstract only might not relate to the search topic directly. Seven of the top 10 articles on TC_2019_ contained search keywords in their abstract only. Typical examples including articles by Li et al. (2000) ranked 1st with TC_2019_ of 1,903, Lim et al. (2001) ranked 3rd with TC_2019_ of 355, Norman et al. (1995) ranked 4th with TC_2019_ of 348, European Food Safety Authority (2017) ranked 6th with TC_2019_ of 322, Macfarlane et al. (1993) ranked 7th with TC_2019_ of 283, Pan et al. (2008) ranked 9th with TC_2019_ of 263, and Hickie et al. (2006) ranked 10th with TC_2019_ of 257. It would be recommended that search keywords in article title or author keywords have more focus on Q fever.  Table 6 shows the top 10 most frequently cited articles with search keywords in their title and author keywords. The citation histories of the Q fever articles ranked top 40th in both TC_2019_ and C_2019_ are shown in Figure 5. These articles contain search keywords in their title or author keywords, were not only highly cited with TC_2019_ ≥ 143 but also high impact in the most recent year of 2019 with C_2019_ ≥ 13. They were summarized as follows:

#### 3.6.1. Comparison of *Coxiella burnetii* Shedding in Milk of Dairy Bovine, Caprine, and Ovine Herds [41]

This article was published by 12 authors from five institutes: National Institute of Agricultural Research (INRA) in France, Adiagene in France, SNGTV in France, La Condamine in France, and Le Bourg in France with TC_2019_ of 157 (ranked 30th) and C_2019_ of 16 (ranked 20th). This study demonstrates the route of shedding of *Coxiella burnetii* in cattle, sheep, and goats. The study revealed that the bacterium is mainly excreted through milk of infected cattle and goats while in sheep it was mainly through faeces and vaginal excretions. The different route of shedding of bacteria may explain the higher association of human outbreaks with sheep as compared to cattle and goats.

#### 3.6.2. Brucellosis and Q Fever Seroprevalences of Nomadic Pastoralists and Their Livestock in Chad [42]

This article was published by seven authors from four institutes: Swiss Tropical Institute in Switzerland, Laboratoire de Recherches Vétérinaires et Zootechniques de Farcha in Chad, Direction de la Planification de la Formation in Chad, and Institute of Veterinary Bacteriology in Switzerland with TC_2019_ of 154 (ranked 32nd) and C_2019_ of 11 (ranked 47th).

This study investigated the association of seropositivity of Q fever in humans and animals in Chad. The authors reported that livestock remained a primary source of Q fever in humans that may be due to consumption of contaminated raw milk or through handling of placenta from infected animals.

#### 3.6.3. The Detection of *Coxiella burnetii* from Ovine Genital Swabs, Milk, and Faecal Samples Using a Single Touchdown Polymerase Chain Reaction [43]

This article was published by three authors from National Institute of Agricultural Research (INRA) in France with TC_2019_ of 143 (ranked 40th) and C_2019_ of 13 (ranked 35th). This study focused on the efficient detection of *Coxiella burnetii* by single touchdown PCR in genital swabs, milk, and faecal samples from infected sheep. It further highlights the importance of its detection from milk and faecal samples.

### 3.7. Research Foci

The top cited articles (Table 6) in Q fever research gave important insights about main research questions such as transmission and shedding routes of *C. burnetii*. Here, a short summary of these articles is given. Livestock plays a key role in maintenance and transmission of *C. burnetii*. However, the route of shedding of bacteria may vary depending on the specie of the animal. Infected cattle and goats shed the bacterium mainly through milk while infected sheep shed the bacterium in faeces and vaginal secretions. This may explain the higher associations of human outbreak of Q fever with sheep populations [41]. Bouvery et al. demonstrated various routes of excretion of *C. burnetii* after experimental infection in goats. *C. burnetii* may be excreted in vaginal secretions till 14 days and 52 days in milk after abortion. However, in faeces, few goats shed *C. burnetii* before abortions and all goats after abortion [44]. Once infected, the goats may experience reproductive problems and shed bacterium in at least two consecutive kidding seasons [45]. The vaginal secretions remain the suitable sample for detection of *C. burnetii* via PCR. On the other hand, milk and faeces may contain certain PCR inhibitory substances. By neutralizing the inhibitory PCR substances in faecal and improved DNA purification from milk samples, the efficacy of PCR may further be improved [43]. Additionally, molecular detection of *C. burnetii* DNA using PCR in clinical samples such as vaginal secretions, faeces, and milk of infected ewes highlights its diagnostic and disease transmission potential [43]. Guatteo et al. used real-time PCR for the detection of *C. burnetii* DNA from milk, faeces, and vaginal secretions of naturally infected cows. They highlighted that only 6% of infected cows shed the bacterium simultaneously through milk, faeces, and vaginal secretions. They also inferred that sampling strategy should consider prevalence and types of samples for serology and shedding [4]. Therefore, identification of DNA from different sample types should be preferred for better diagnosis of the disease. Guatteo et al. further demonstrated that infected cows may be sporadic or persistent shedder of *C. burnetii*. The persistent shedders are highly seropositive as compared to sporadic shedders. Therefore, antibody monitoring through serological assays such as ELISA could be an effective tool to identify the persistent and heavy shedders [46].

Furthermore, Schelling et al. demonstrated that human Q fever infection is associated with consumption of raw milk and direct contact with placenta of infected animals [42]. The association of human outbreak with infected animals was further reported while investigating an outbreak of Q fever in Netherland [47]. The higher incidence of Q fever in human was reported in areas with higher sheep densities and wind speeds which may speculate the higher aerosol transmission [48].

Word distribution in article title, abstract, author keywords, and *KeyWords Plus* represents the most important information in conveying the findings of a study. Therefore, an analysis of word distribution can be very useful to evaluate the trends in a particular research field [49]. In the last decade, to determine research foci and trends during different years, Ho's group proposed distributions of article titles and abstracts, author keywords, and *KeyWords Plus* [49]. These analyses could minimize various limitations, such as the incomplete meaning of single words in article title and abstract, small sample size for author keywords, and the indirect relationship between *KeyWords Plus* and the research topics [50]. Therefore, these four kinds of words (in article titles, article abstracts, author keywords, and *KeyWords Plus*) were examined during the designated period to show the rough trends while minimizing the year-to-year fluctuations [49]. Distribution of words in article titles, article abstract, author keywords, and *KeyWords Plus* allowed us to visualize the major trends and foci in research related to Q fever over the years.

The 20 most frequently used author keywords of four subperiods (1990s, 2000s, and 2010s) are listed in Table 7. The most frequently used author keywords, except for the searching words, “Q fever” and “*Coxiella burnetii*,” were seroprevalence, zoonosis, and ELISA. Overall, this table depicts that the most used words are related to the major focus of researchers working in the following fields related to Q fever: zoonosis/zoonoses (transmission of an infectious disease from animal to human), seroprevalence (measure of the disease burden through serological investigation), laboratory diagnosis (ELISA and PCR), clinical manifestations (abortion, endocarditis), vector (ticks), and hosts (sheep, goat and cattle). These topics are discussed below along with the results of word cluster analyses.

A word cluster analysis was also performed to identify the potential research hotspots in this topic. For this, synonymic expressions/words from words analyses denoting to a particular term were summed up. Our findings revealed that major focus of research about Q-fever dealt with its diagnostics, determination of host range, and clinical manifestation of the disease. We further investigated the keywords used in diagnostics, host range, and clinical manifestation that could facilitate to have an idea the most used keywords during different periods in each discipline.

**Figure 6:**
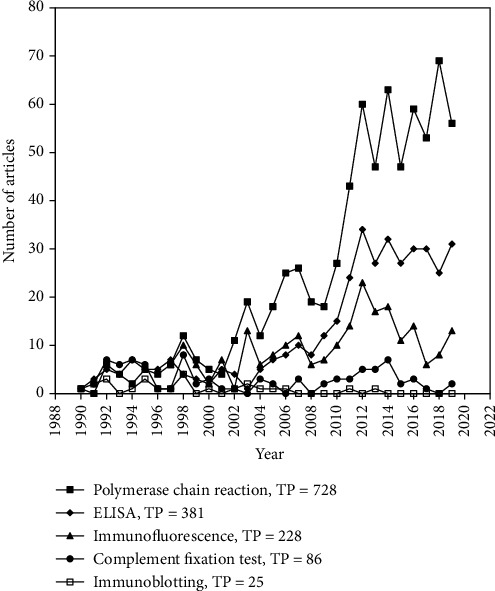
Research trends related to diagnostic techniques in Q fever.

The most used cluster of keywords in terms of diagnostics include polymerase chain reaction (based-PCR, broad range PCR, bulk milk PCR test, c-PCR, conventional PCR, culture-PCR, dPCR, fret-qPCR, icc-PCR, immuno-PCR, iPCR, irs-PCR, lcn-PCR, linear-PCR, m-PCR, monoazide-PCR, mPCR, mPCR1, mPCR2, mPCR3, mPCR4, mPCR5, mpn-PCR, multiplex PCR, nested PCR, nested trans-PCR, nested-PCR, omp-PCR, PCR, PCR assay, PCR detection, PCR-amplification, PCR-based assays, PCR-detection, PCR-dgge, PCR-electrospray, PCR-ELISA, PCR-enzyme-linked, PCR-esi-ms, PCR-response, PCR-restriction, PCR-reverse, PCR-rlb, PCR-screened, PCR, PCR-rlbs, PCRs, polymerase chain reaction (PCR), polymerase chain reaction-restriction fragment length polymorphism (PCR-RFLP), PCR-rflp, polymerase chain reaction, q fever PCR, q-PCR, qPCR,, qPCRs, qrt-PCR, quantitative PCR, quantitative real-time PCR, real time PCR, real-time PCR, real-time qPCR, real-time-PCR, rrt-PCR, rt-PCR, rt-qPCR, rti-PCR, single-tube nested PCR, site-PCR, taqman real-time PCR, touchdown-PCR, trans-PCR, transcriptase-PCR, transPCR, two-step broad-range PCR, whole genome PCR scanning, xl PCR) (TP = 728), ELISA (capture-ELISA, ELISAs, ELISA, capture-ELISA, i-ELISA, IgM-ELISA, mona-ELISA, p-ELISA, antibody-ELISA, cELISA, burnetii-ELISA, phase-specific ELISA, capture ELISA, immunosorbent-assay ELISA, ELISA test, capture ELISA, immunosorbent-assay ELISA, enzyme-linked-immunosorbent-assay, enzyme-linked immunosorbent assay, ovine enzyme-linked immunosorbent assay, enzyme linked immunosorbent assay, enzyme-linked-immunosorbent, enzyme-linked-immunosorbent-assay) (TP = 381), immunofluorescence (TP = 228), complement fixation test (Complement fixation, complement-fixation, complement-fixation tests, Micro-CFT, CFT) (TP = 86), and immunoblotting (TP = 25) as shown in Figure 6. During the last decade, PCR, a technique for molecular characterization, emerged as the most widely used keyword in literature followed by ELISA, a serological based diagnostic approach, representing these techniques as the major research hotspot. However, the use of other serological techniques such as complement fixation test and immunoblotting as keyword in Q fever research remains limited during the same time period. It should be noted that in infectious diseases, isolation and identification of causative agent remain the gold standard for diagnosis of the disease. However, the difficulty of culturing *Coxiella burnetii* in laboratory conditions and requirement of stringent biosafety level (BSL-3) facility necessitate the use of indirect diagnostic techniques such as PCR and ELISA.

**Figure 7:**
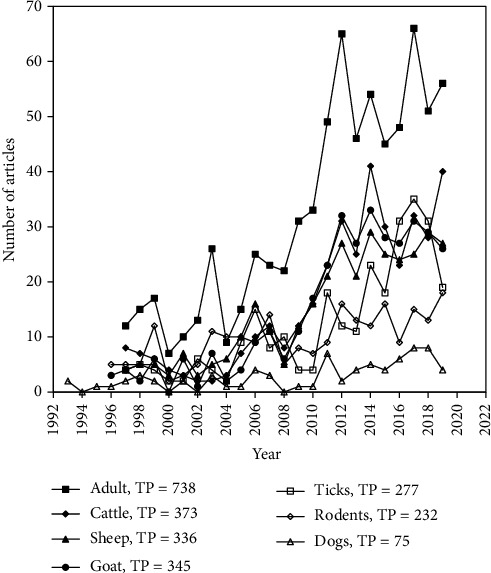
Research trends related to hosts in Q fever.

To effectively control any disease, it is pertinent to know the range of hosts of the infectious agent, which play pivotal role in maintenance and transmission of the agent. The cluster of keyword analysis showed that human (adult, women, adults, children, human, men) is the most widely used keyword (TP = 738) followed by cattle (cows, cattle, dairy cattle, dairy-cattle) (TP = 373), goat (goat, caprine) (345), sheep (ovine, sheep, ewes) (TP = 336), and ticks (tick, ticks, ixodes, rhipicephalus, ricinus, haemaphysalis, hyalomma, ixodes-ricinus ticks, amblyomma) (TP = 277), respectively. The other keywords in host (Figure 7) used were rodents (rodents, murine, mouse, mice, rats) (TP = 232), dog (TP = 75), cats (TP = 62), wildlife (TP = 54), and pigs (TP = 49). Q fever, a zoonotic disease, can be transmitted from animal to animal and animal to human directly or indirectly through ticks [51]. However, the *C. burnetii* infected animals such as cattle, sheep, and goats mainly transmit the infectious agent to animal holders or abattoir workers.

**Figure 8:**
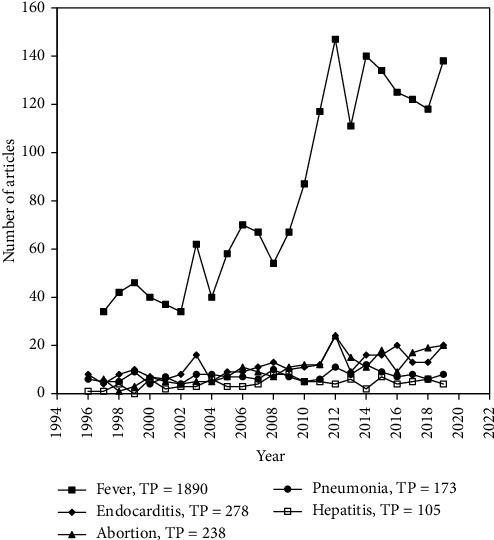
Research trends related to clinical manifestations of Q fever.

For clinical diagnosis, it is of paramount importance to know the clinical manifestations of a particular disease for its effective diagnosis. Therefore, a cluster word analysis for clinical manifestations of Q fever was performed. In cluster word analysis, top 5 clinical manifestations (Figure 8) were fever (TP = 1890), endocarditis (TP = 278), abortion (abortion, abortions) (TP = 238), pneumonia (pneumonia, community acquired pneumonia) (TP = 173), and hepatitis (TP = 105). Collectively, the data shows that most extensively used keyword in clinical manifestations remains endocarditis, abortion, and pneumonia.

Therefore, *Coxiella burnetii* may be considered as a potential causative agent in patients with clinical presentation of endocarditis, abortion, or pneumonia which may improve diagnosis and treatment. Involvement of animals as a potential source for human Q fever infections requires better coordination between veterinarians and human physicians. Such coordinated effort from multidiscipline researchers will contribute to a better understanding of the distribution in other animals. Similarly, determination of associated risk factors in transmission and development of disease will pave the way for development of better preventive and therapeutic approaches.

## 4. Conclusion

This bibliometric analysis gives an insight on the developments in the discipline of Q fever, and it also provided the details of most influential publications, institutes, countries, and authors. Last decade showed the highest number of publications. Additionally, publications relevant to Q fever were published from the USA, Europe, and Australia. That could be helpful for researchers to collaborate with the relevant research groups and can further help post-docs or PhD for those pursuing their careers in Q fever research. *PLoS One* remained the most productive journal followed by “Infection and Immunity” and “Clinical and Infectious Diseases.” This demonstrates the importance of these journals in research relevant to Q fever and can be helpful for authors looking to publish similar research. Furthermore, research trends and hot areas including the clinical presentation, diagnosis, and host of Q fever were identified. Endocarditis, abortion, and pneumonia remained the most commonly used keywords to demonstrate clinical presentation. The use of PCR and ELISA, techniques used for the diagnosis, were most used over the recent years. In the end, we identified keywords pertinent to species infected with Q fever. This showed that human remained the most commonly used keyword followed by cattle and sheep. The presence of Q fever in human as well as various animal species such as cattle, sheep, and goat demonstrates its zoonotic importance further highlights the importance of collaborative effort from human physicians and veterinarians under the umbrella of one health to mitigate Q fever.

## Figures and Tables

**Table 1:** Citations and authors according to document type.

Document type	TP	%	TP^∗^	AU	APP	TC_2019_	CPP_2019_
Article	2840	77	2837	16948	6.0	57664	20
Letter	221	6.0	221	883	4.0	1335	6.0
Review	217	5.9	217	837	3.9	11531	53
Meeting abstract	206	5.6	203	1068	5.3	271	1.3
Proceedings paper	139	3.8	139	662	4.8	2293	16
Editorial material	80	2.2	78	245	3.1	561	7.0
Note	72	2.0	72	346	4.8	1005	14
News item	24	0.65	10	11	1.1	58	2.4
Book chapter	23	0.63	23	56	2.4	312	14
Correction	9	0.25	9	46	5.1	2	0.22
Addition correction	2	0.054	2	7	3.5	0	0
Reprint	2	0.054	1	16	16	0	0

TP: number of publications; TP^∗^: number of publications with author information; AU: number of authors; APP: number of authors per publication; TC_2019_: the total number of citations from Web of Science Core Collection since publication year to the end of 2019; CPP_2019_: number of citations (TC_2019_) per publication (TP).

**Table 2:** The top 10 most productive journals.

Journal	TP (%)	IF_2019_	APP	CPP_2019_	Web of Science category
PLoS One	104 (3.7)	2.74	7.5	15	Multidisciplinary sciences
Infection and Immunity	89 (3.1)	3.201	5.1	37	Immunology, infectious diseases
Vector-Borne and Zoonotic Diseases	66 (2.3)	2.249	7.7	12	Public, environmental and occupational health, infectious diseases
Clinical Infectious Diseases	63 (2.2)	8.313	6.0	76	Immunology, infectious diseases, microbiology
American Journal of Tropical Medicine and Hygiene	56 (2.0)	2.126	7.9	20	Public, environmental and occupational health, tropical medicine
Epidemiology and Infection	54 (1.9)	2.152	6.4	20	Public, environmental and occupational health, infectious diseases
Acta Virologica	52 (1.8)	0.793	4.6	8.6	Virology
Journal of Clinical Microbiology	51 (1.8)	5.897	6.6	52	Microbiology
European Journal of Epidemiology	45 (1.6)	7.135	4.6	22	Public, environmental and occupational health
Emerging Infectious Diseases	39 (1.4)	6.259	7.2	38	Immunology, infectious diseases

TP: number of publications; IF_2019_: journal impact factor in 2019; APP: number of authors per publication; CPP_2019_: number of citations (TC_2019_) per publication (TP).

**Table 3:** Top 10 productive countries.

Country	TP	TP R (%)	IP R (%)	CP R (%)	FP R (%)	RP R (%)	SP R (%)	CPP_2019_
USA	633	1 (22)	1 (20)	1 (33)	1 (18)	1 (19)	1 (32)	29
France	520	2 (18)	2 (16)	2 (26)	2 (15)	2 (15)	2 (15)	30
Netherlands	279	3 (10)	3 (9.0)	3 (13)	3 (8.8)	3 (8.9)	N/A	16
Germany	208	4 (7.4)	4 (6.6)	5 (10)	4 (6.1)	4 (6.0)	4 (5.4)	16
Spain	182	5 (6.4)	5 (6.2)	7 (7.4)	5 (5.7)	5 (5.9)	7 (3.3)	16
Australia	152	6 (5.4)	6 (4.9)	8 (7.1)	6 (4.6)	6 (4.5)	7 (3.3)	18
UK	124	7 (4.4)	9 (2.7)	4 (11)	8 (2.8)	8 (2.8)	4 (5.4)	26
Slovakia	106	8 (3.8)	10 (2.3)	6 (9.0)	10 (2.7)	9 (2.7)	12 (1.1)	19
Italy	94	9 (3.3)	7 (2.9)	11 (4.8)	7 (2.9)	7 (3.0)	12 (1.1)	19
Japan	89	10 (3.2)	8 (2.9)	14 (4.1)	9 (2.7)	10 (2.6)	12 (1.1)	20

TP: number of total articles; IP: independent articles; CP: internationally collaborative articles; FP: first-author articles; RP: corresponding-author articles: SP: single-author articles; R: rank; CPP_2019_: number of citations (TC_2019_) per publication (TP); N/A: not available.

**Table 4:** Top 10 productive institutions with six publication indicators and their CPP_2019_.

Institute	TP	TP R (%)	IP R (%)	CP R (%)	FP R (%)	RP R (%)	SP R (%)	CPP_2019_
Aix Marseille University, France	104	1 (3.7)	6 (1.7)	2 (4.6)	2 (2.6)	3 (1.9)	N/A	12
Jeroen Bosch Hospital, Netherlands	97	2 (3.4)	67 (0.22)	1 (5)	12 (0.85)	13 (0.77)	N/A	18
Slovak Academy of Sciences, Slovakia	95	3 (3.4)	2 (3.8)	6 (3.1)	3 (2.3)	2 (2.4)	N/A	20
University of Mediterranee, France	92	4 (3.3)	1 (4.2)	7 (2.8)	1 (2.7)	1 (2.5)	N/A	51
Radboud University of Nijmegen, Netherlands	83	5 (2.9)	18 (0.76)	3 (4.0)	5 (1.5)	5 (1.4)	N/A	12
Centers for Disease Control and Prevention, USA	78	6 (2.8)	6 (1.7)	5 (3.3)	7 (1.2)	7 (1.3)	N/A	33
National Institute of Allergy and Infectious Diseases, USA	76	7 (2.7)	3 (3.7)	10 (2.2)	4 (1.7)	3 (1.9)	2 (3.3)	39
National Institute for Public Health and the Environment, Netherlands	74	8 (2.6)	114 (0.11)	4 (3.8)	9 (0.92)	10 (0.88)	N/A	20
National Institute of Agricultural Research (INRA), France	60	9 (2.1)	12 (1.1)	8 (2.6)	8 (1.0)	8 (1.0)	2 (3.3)	29
Faculte de Medecine Marseille, France	59	10 (2.1)	5 (2.1)	12 (2.1)	6 (1.3)	5 (1.4)	9 (1.1)	57

TP: total number of highly cited articles; TPR (%), IPR (%), CPR (%), FPR (%), RPR (%), and SPR (%): the rank and percentage of total articles, single institution articles, interinstitutionally collaborative articles, first-author articles, corresponding-author articles, single-author articles in their total articles; CPP_2019_: number of citations (TC_2019_) per publication (TP); N/A: not available.

**Table 5:** Top 10 most productive authors.

Author	Rank (TP)	Rank (FP)	Rank (RP)	*h*	Rank (*j*)
D. Raoult	1 (284)	1 (16)	1 (117)	1.435	1 (133)
J.L. Mege	2 (70)	N/A	3 (37)	*π*/2	3 (37)
R.A. Heinzen	3 (66)	35 (6)	2 (46)	1.549	2 (47)
W. van der Hoek	4 (53)	4 (11)	8 (14)	0.9048	5 (25)
J.E. Samuel	5 (52)	420 (1)	4 (29)	1.536	4 (30)
P.C. Wever	6 (47)	N/A	175 (2)	*π*/2	397 (2)
P.M. Schneeberger	7 (43)	420 (1)	360 (1)	0.7854	397 (2)
C. Capo	8 (42)	22 (7)	175 (2)	0.3805	103 (7)
C.P. Bleeker-Rovers	9 (41)	N/A	113 (3)	*π*/2	308 (3)
H. Lepidi	10 (34)	182 (2)	360 (1)	0.4636	308 (3)

TP: total number of articles; FP: number of first-author articles; RP: number of corresponding-author articles; *h*: *Y*-index constant, publication characteristics; *j*: *Y*-index constant, publication potential; N/A: not available.

**Table 6:** The top ten most frequently cited articles with search keywords in their title and author keywords.

Rank (TC_2019_)	Rank (C_2019_)	Title	Country	Reference
30 (157)	20 (16)	Comparison of *Coxiella burnetii* shedding in milk of dairy bovine, caprine, and ovine herds	France	[41]
32 (154)	47 (11)	Brucellosis and Q-fever seroprevalences of nomadic pastoralists and their livestock in Chad	Switzerland, Chad	[42]
40 (143)	35 (13)	The detection of *Coxiella burnetii* from ovine genital swabs, milk and fecal samples by the use of a single touchdown polymerase chain reaction	France	[43]
43 (137)	144 (6)	Hyperendemic focus of Q fever related to sheep and wind	France	[48]
49 (127)	90 (8)	Experimental *Coxiella burnetii* infection in pregnant goats: excretion routes	France	[44]
56 (121)	90 (8)	Shedding routes of *Coxiella burnetii* in dairy cows: Implications for detection and control	France	[4]
83 (98)	90 (8)	*Coxiella burnetii* shedding by dairy cows	France	[46]
103 (88)	217 (5)	Investigation of a Q fever outbreak in a rural area of The Netherlands	Netherlands	[47]
103 (88)	217 (5)	Goats may experience reproductive failures and shed *Coxiella burnetii* at two successive parturitions after a Q fever infection	France	[45]
110 (87)	217 (5)	Effect of vaccination with phase I and phase II *Coxiella burnetii* vaccines in pregnant goats	France	[52]

TC_2019_: the total number of citations from Web of Science Core Collection since publication year to the end of 2019; C_2019_: the number of citations of an article in 2019 only.

**Table 7:** The 20 most frequently used author keywords.

Author keywords	TP	1990-2019 R (%)	1990-1999 R (%)	2000-2009 R (%)	2010-2019 R (%)
*Coxiella burnetii*	881	1 (51)	1 (37)	1 (55)	1 (55)
Q fever	706	2 (41)	2 (25)	2 (45)	2 (45)
*Coxiella*	94	3 (5.5)	8 (3.6)	7 (5.4)	4 (6.2)
Seroprevalence	91	4 (5.3)	48 (0.90)	11 (4.6)	3 (6.7)
Zoonosis	84	5 (4.9)	13 (2.7)	6 (5.5)	8 (5.4)
Elisa	79	6 (4.6)	13 (2.7)	9 (5.2)	9 (5.2)
Cattle	77	7 (4.5)	8 (3.6)	33 (1.3)	5 (5.8)
Serology	77	7 (4.5)	6 (4.1)	20 (2.1)	6 (5.5)
Epidemiology	76	9 (4.4)	8 (3.6)	3 (6.2)	12 (4.4)
Sheep	76	9 (4.4)	18 (2.3)	3 (6.7)	11 (4.6)
Zoonoses	76	9 (4.4)	21 (1.8)	15 (3.3)	6 (5.5)
PCR	72	12 (4.2)	48 (0.9)	7 (5.5)	10 (4.7)
Abortion	64	13 (3.7)	26 (1.4)	3 (5.8)	13 (3.7)
Q fever	63	14 (3.7)	4 (12)	14 (3.6)	18 (2.2)
Endocarditis	58	15 (3.4)	13 (2.7)	9 (4.7)	15 (3.3)
Ticks	52	16 (3.0)	26 (1.4)	17 (2.2)	14 (3.6)
Rickettsia	47	17 (2.7)	8 (3.6)	11 (4.4)	18 (2.2)
Goat	44	18 (2.6)	18 (2.3)	16 (2.6)	16 (2.6)
Coxiella burnetii	41	19 (2.4)	3 (18)	N/A	N/A
Polymerase chain reaction	32	20 (1.9)	6 (4.1)	60 (0.73)	24 (1.8)

TP: number of articles; R: rank in a period; N/A: not available.

## Data Availability

The data that support the findings of this study are available from the corresponding author upon reasonable request.
